# Recent updates on antidiabetic and antiobesity potential of carnosic acid

**DOI:** 10.17179/excli2021-4259

**Published:** 2021-09-28

**Authors:** Diya S. Nair, Digumarthy Niharika, Aishwariya Madhavan, Shweta Sharma, Apurva Kumar Ramesh Joshi

**Affiliations:** 1 Department of Biochemistry, School of Sciences (B1), Jain (Deemed to be University), JC Road, Bangalore, Karnataka, India 560041

## ⁯⁯⁯⁯

***Dear Editor,***

Rosemary (*Rosmarinus officinalis*) extracts have been extensively studied for their ability to ameliorate traits of metabolic dyshomeostasis (Sedighi et al., 2015[19]; Naimi et al., 2017[11]). Carnosic acid, ursolic acid and rosmarinic acid are among the major bioactives of the herb (Li et al., 2019[8]). Carnosic acid (CA) is a diterpene that is known for antidiabetic (summarized in Table 1(Tab. 1); References in Table 1: Hasei et al., 2021[3]; Lee et al., 2018[7]; Lipina and Hundal, 2014[9]; Naimi et al., 2017[10]; Ou et al., 2017[12], 2018[13]; Park and Mun, 2013[14]; Park and Sung, 2015[15]; Razavi et al., 2020[17]; Song et al., 2018[21]; Tsai et al., 2014[23]; Wang et al., 2011[25], 2019[24]; Xia et al., 2017[27]; Xie et al., 2017[28], 2018[29]; Zhao et al., 2015[31]), antiobesity (summarized in Table 1(Tab. 1)), antioxidant (Huang et al., 1996[5]; Sahu et al., 2014[18]; Birtić et al., 2015[2]; Thummuri et al., 2017[22]), and neuroprotective (Azad et al., 2011[1]; Hou et al., 2013[4]; Wu et al., 2015[26]) properties. Rosemary is used as source material for preparation of CA-enriched extracts for commercial applications as the herb is known for having high levels of CA (in excess of 2 %). Owing to its antioxidant potential, CA-rich rosemary extracts have now been approved for use as a food additive (E392) (Younes et al., 2018[30]).

Rosemary or rosemary-derived preparations have been demonstrated to modulate glycemic parameters in human subjects. Consumption of rosemary tea for 90 days has been reported to reduce glycated hemoglobin levels in addition to alleviating insulin resistance in type 2 diabetes subjects (Quirarte-Báez et al., 2019[16]). Reduction in blood glucose levels has been reported following 4-week consumption of rosemary leaf powder (Labban et al., 2014[6]). Similarly, consumption of rosemary powder (3 g/day) for 8 weeks has been reported to decrease glucose and glycated hemoglobin levels in type 2 diabetes patients receiving either metformin or glucomid (Shawabkeh and Jamal, 2017[20]). Considering that CA is abundantly found in rosemary, it is not surprising that the diterpene has been explored for its antidiabetic and antiobesity effects. Table 1(Tab. 1) summarizes experimental reports demonstrating antidiabetic and antiobesity effects of CA. In view of the status of CA-enriched extracts of rosemary as an approved food additive and known antidiabetic and antiobesity effects, we opine that CA has the potential to be investigated for antidiabetic effects in clinical settings.

| | **Summary of the study** | **Reference** |
|---|---|---|
| 1. | Hasei et al. studied the effect of CA on expression of gluconeogenic genes *in vitro*. CA treatment of HEPG2 cells was associated with abrogation of forskolin-induced up-regulation of phosphoenolpyruvate carboxykinase and glucose-6-phosphatase expression. Further, CA treatment was associated with increase in the magnitude of AMP-activated protein kinase (AMPK) and acetyl CoA carboxylase (ACC) phosphorylation. | Hasei et al., 2021 |
| 2. | Razavi et al. evaluated the effect of CA on metabolic abnormalities in rats intraperitoneally treated with olanzapine for 14 days. Treatment of rats with CA was efficient in reducing the body weight gain and systolic pressure in olanzapine-treated rats, in addition to reducing glucose and triglyceride levels in plasma. Olanzapine alone reduced the extent of AMPK and ACC phosphorylation in liver. CA-treatment was found to be associated with enhanced phosphorylation of hepatic AMPK and ACC, indicating that the metabolic rearrangements caused by activation of AMPK may be an important participant in the observed protective effects of CA. | Razavi et al., 2020 |
| 3. | The study of Wang et al. showed that CA exerted competitive inhibition of amylase and non-competitive inhibition of glucosidase. CA was also reported to suppress the area under the curve for glucose response in mice after oral maltose or starch challenge. | Wang et al., 2019 |
| 4. | Lee et al. assessed the ability of dietary CA (0.02 %) to modulate lipogenic mechanisms in ovariectomized mice fed high fat diet. Dietary administration of CA was associated with reduced body weight gain and reductions in retroperitoneal, perirenal fat, gonadal fat, mammary fat pad and mesenteric fat weight. High fat diet-induced hyperinsulinemia and hyperleptinemia as well as increased serum triglyceride and free fatty acid levels were normalized in ovariectomized mice as a result of incorporation of CA in the diet. Extent of AMPK and ACC phosphorylation and expression of Peroxisome proliferator-activated receptor (PPAR)-alpha and carnitine palmitoyltransferase-1 (CPT-1) was higher in livers of ovariectomized mice receiving CA, which also lowered the expression of SREBP1c and fatty acid synthase. Further, dietary administration of CA was associated with reduced adipocyte area and reduced expression of tumor necrosis factor-alpha and interleukin-6 in adipose tissue of ovariectomized mice. | Lee et al., 2018 |
| 5. | Xie et al. carried out *in vitro* (cell-based) and *in vivo* (with type 1 and type 2 diabetic experimental models) assessments to delineate whether CA could prevent diabetes-induced neuropathy. Using mesangial cells subjected to glucotoxicity, investigators observed that CA-treated cells exhibited augmented expression of Nrf2-controlled genes and suppression of genes controlled by NF-κB. CA-treatment caused increased nuclear accumulation of Nrf2 with concomitant increase in expression and protein levels of hemoxygenase-1. Nuclear accumulation of p65 was reduced in CA-treated cells along with lowered expression of MCP1, TNF-alpha, iNOX and COX-2. The monogenic db/db mice were used as a type 2 diabetes model to evaluate whether CA could ameliorate neuro- pathy. CA treatment lowered circulating glucose, triglycerides, total cholesterol and LDL-cholesterol in db/db mice, while improving tolerance to glucose and sensitivity to insulin. Further water consumption and urinary volumes were reduced in db/db mice along with augmented urinary creatinine excretion. The antidiabetic effects of CA were found to be associated with amelioration of neuropathy in db/db mice. CA-treatment led to reduced albumin excretion and reduced albumin to creatinine ratio. Histologically, lower degree of glo- | Xie et al., 2018 |

EXCLI Journal 2021;20:1476-1481 – ISSN 1611-2156
Received: September 03, 2021, accepted: September 13, 2021, published: September 28, 2021

| | | |
|---|---|---|
| | merulosclerosis was evident in CA-treated db/db mice. Interestingly, CA-treatment was also effective in ameliorating diabetes and neuropathy in mice treated with streptozotocin. CA-treatment led to reduction in blood glucose, relative kidney weights, urinary output, albumin excretion and urinary albumin to creatinine ration in diabetic mice. Improvement in kidney architecture, as evidenced by reduced glomerular basement membrane thickness and reduced glomerulosclerosis score, was observed in streptozotocin treated mice administered CA, along with up-regulated expression of hemoxygenase1. | |
| 6. | Anti-diabetic effects of CA were studied in a diabetic rat model (high fat feeding + streptozotocin). Treatment of diabetic rats with CA (30 mg/kg b.w.) elicited reduction in blood glucose, insulin, total cholesterol, triglyceride levels, and activities of creatine kinase and aspartate aminotransferase. Further, CA reduced the extent of oxidative damage in liver, heart and kidney as evidenced by normalization of lipid peroxidation and other parameters. Expression of MCP1, TNF-alpha and NF-κB (p65) was reduced in abdominal aorta of diabetic rats treated with CA. Prebiotic-like effects of CA were observed as evidenced by its ability to facilitate greater magnitude of prevalence of diabetes resistant bacteria in the gut microflora. | Ou et al., 2018 |
| 7. | Song et al. studied the role of MARCKS (myristoylated alanine-rich C-kinase substrate) in the ability of CA to alleviate non-alcoholic fatty liver disorder in high fat diet fed mice. Oral treatment with CA reduced inflammatory index, extent of oil-red O staining, triglyceride and cholesterol content while increasing MARCKS+ve and PPAR-alpha+ve cell count in liver. Further, CA-treatment effectively lowered levels glucose, insulin, aspartate aminotransferase, alanine aminotransferase and several cytokines in serum. Expression of MARCKS and PPAR-alpha was increased in livers of CA-treated mice, while that of SREBP-1C, fatty acid synthase, acetyl co-A carboxylase, and SCD1 were down regulated. Exacerbation of non-alcoholic fatty liver disorder as evidenced by molecular and biochemical analysis in MARCKS-deficient mice provided mechanistic involvement for role of MARCKS in alleviation of non-alcoholic fatty liver disorder in high fat diet fed mice treated with CA. | Song et al., 2018 |
| 8. | Exposure of L6 cells to CA elicited increase in basal and insulin-mediated deoxyglucose uptake. Mechanistic studies revealed that CA-induced glucose uptake was attributable to AMP-activated protein kinase as revealed by increased AMPK and acetyl Coa carboxylase phosphorylation and inhibitory effect of compound C (AMPK inhibitor) CA-induced deoxyglucose uptake. | Naimi et al., 2017a |
| 9. | Exposure of hepatocytes to CA resulted in lower levels of nuclear accumulation of mature SREBP and reduced expression of key SREBP target genes. CA-treated hepatocytes accumulated lower levels of cholesterol and triglycerides. Treatment with CA ameliorated hyperlipidemia and reduced blood glucose and insulin levels in obese mice. Mechanistic studies revealed that the above metabolic effects of CA may be mediated by proteasomal degradation of mature SREBP. | Xie et al., 2017 |
| 10. | CA was found to inhibit formation of fluorescent advanced glycated end products in models involving bovine serum albumin and glucose, glyoxal and methylglyoxal. Further, treatment with CA resulted in formation of lower levels of glyoxal and methylglyoxal in BSA/glucose model and lower levels of caboxymethyl lysine and carboxyethyl lysine in above said models. | Ou et al., 2017 |
| 11. | Protective effects of CA were evaluated in db/db diabetic mice subjected to induction of arthritis by intradermal injection of collagen. CA treatment led to reduction in glucose levels in diabetic and diabetic-arthritic mice along with improved tolerance to glucose and insulin. Compared to the respective controls, CA treatment reduced IL-17, IL-1 | Xia et al., 2017 |

| | | |
|---|---|---|
| | beta, TNF-alpha, Receptor activator of nuclear factor kappa-B ligand (RANKL), MIP-1, IL-6 and IFN-gamma in serum of diabetic and diabetic-arthritic mice. Enzymatic anti-oxidant defenses were up-regulated in CA-treated mice, resulting in lower levels of reactive oxygen species and lipid peroxidation. Interestingly, administration of CA abrogated traits of arthritis in diabetic-arthritic mice as evidenced by reductions in cumulative arthritis incidence, arthritic edema, RANKL +ve cells in metatarsophalangeal joints, arthritic score and serum Ig G anti-collagen. Mechanistic studies also revealed that CA possessed propensity to prevent osteoclastogenesis. | |
| 12. | Zhao et al. evaluated the antidiabetic effects of CA-enriched rosemary extract (CA-RE) supplemented as a part of diet to high-fat fed mice for 16 weeks. CA-RE elicited lower magnitude of body weight gain and reduced fat mass in mice fed-high fat diet. Further, CA-RE treatment was associated with lower glucose, insulin, alanine aminotransferase activity, aspartate aminotransferase activity, malondialdehyde and TNF-alpha levels in the plasma of high-fat diet fed mice. Further, CA-RE treated mice fed high-fat diet showed lower level of triglyceride, non-esterified free fatty acids, advanced glycated end products and receptor for advanced glycated end products in liver as compared to that of high-fat diet controls. | Zhao et al., 2015 |
| 13. | The monogenic diabetic ob/ob mice were fed CA in diet (0.1 and 0.02 %) for assessment of anti-obesity potential of the diterpene. Incorporation of CA in the diet resulted in reduced body weight gain, reduced feed intake and reduced fat content, in addition to reducing serum insulin, triglyceride and total cholesterol levels. The CA-treated ob/ob mice exhibited improved tolerance towards intraperitoneal glucose. Gene expression studies revealed that the effects of CA were mediated by reduced expression of L-fatty acid binding protein, SCD1 and fatty acid synthase and up-regulation of carnitine palmitoyltransferase-1. Levels of TNF-alpha, IL-6 and MCP1 were reduced in the serum of ob/ob mice treated with CA. | Park and Sung, 2015 |
| 14. | CA was reported to attenuate TNF-alpha-mediated suppression of glucose uptake in 3T3-L1 adipocytes *in vitro*. The permissive effect of CA on glucose uptake was associated with reversal of reductions in tyrosine phosphorylation of insulin receptor substrate and serine phosphorylation of protein kinase B. Exposure of 3T3-L1 cells to TNF-alpha led to increased expression of IL-6 and MCP1 and increased phosphorylation of ERKs and JNKs. These changes were abrogated by exposure of TNF-alpha-treated cells to CA. Mechanistic studies revealed that CA was effective in suppressing activation of NF-κB pathway induced by TNF-alpha. Further, CA exposure was found to restore expression of adiponectin and PPAR-gamma in cells exposed to TNF-alpha. | Tsai et al., 2014 |
| 15. | Lipina and Hundal evaluated the mechanism of anti-diabetic effects of CA by assessing its effects on glucose uptake in L6 myotubes. Exposure of myotubes to CA was associated with increased glucose uptake (independent of insulin) and increase in GLUT4 levels in plasma membrane preparations, indicating that CA may exert insulin-mimetic effects. CA-induced up-regulation of glucose uptake was not affected by PI3 Kinase, PPAR-gamma and PPAR-alpha antagonists. CA-treated cells exhibited elevated phosphorylation of PKB and AMPK. However, AMPK activation was not found to be responsible CA-induced increase in glucose uptake, as revealed by lack of effect AMPK knockdown on glucose uptake in CA-treated cells. Immunoblotting technique revealed that CA-treatment was associated with increase in the levels of demethylated PP2A catalytic subunit and treatment of cells with an inhibitor of protein phosphatase methylesterase-1 reduced both the levels of demethylated PP2A catalytic subunit and glucose uptake, indicating | Lipina and Hundal, 2014 |

| | | |
|---|---|---|
| | that suppression of the protein phosphatase activity towards PKB is responsible for CA-induced up-regulation of glucose uptake in myotubes. | |
| 16. | Dietary administration of CA (0.05 % of diet) to ob/ob mice for 5 weeks resulted in reduced weight gain, without affecting food intake. Magnetic resonance pictures suggested CA treatment was associated with lower burden of visceral fat mass. In addition, CA-treated mice exhibited lower levels of glucose, triglyceride, total cholesterol, free fatty acids and alanine aminotransferase activity in serum. Glucose tolerance was improved as a result of CA-treatment with concomitant reduction in hepatic fat content in mice. | Wang et al., 2011 |
| 17. | Park and Mun observed that dietary CA alleviated steatosis in high fat diet fed mice. The mechanism responsible for the observed effect was mainly attributed to reduced expression of genes involved in lipogenesis and increased expression of genes related to beta-oxidation. Treatment with CA improved glucose tolerance and alleviated insulin resistance in high fat diet fed mice with concomitant decrease in serum insulin, free fatty acids, and triglyceride and total cholesterol levels. With regards to gene expression, treatment with CA resulted in suppression of SREBP-1c, steroyl co-A desaturase and fatty acid synthase expression, while that of PPAR-alpha, carnitine palmitoyltransferase and acyl co-A oxidase was up-regulated. | Park and Mun, 2013 |

***Acknowledgments***

***Conflict of interest***

None.

## Acknowledgements

Authors are thankful to Jain (Deemed to be University), Bangalore for the support.

## Conflict of interest

None.

## Figures and Tables

**Table 1:**
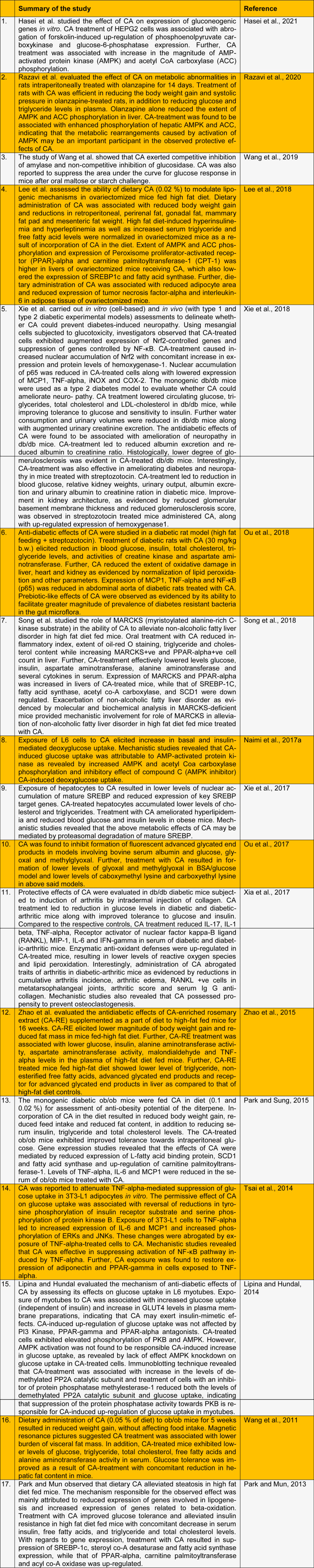
Summary of experimental reports demonstrating metabolic effects of carnosic acid
